# Rehabilitation Using Mobile Health for Older Adults With Ischemic Heart Disease in the Home Setting (RESILIENT): Protocol for a Randomized Controlled Trial

**DOI:** 10.2196/32163

**Published:** 2022-03-03

**Authors:** John A Dodson, Antoinette Schoenthaler, Greg Sweeney, Ana Fonceva, Alicia Pierre, Jonathan Whiteson, Barbara George, Kevin Marzo, Wendy Drewes, Elizabeth Rerisi, Reena Mathew, Haneen Aljayyousi, Sarwat I Chaudhry, Alexandra M Hajduk, Thomas M Gill, Deborah Estrin, Lara Kovell, Lee A Jennings, Samrachana Adhikari

**Affiliations:** 1 Geriatric Cardiology Program, Medicine and Population Health Leon H Charney Division of Cardiology NYU Grossman School of Medicine New York, NY United States; 2 Department of Population Health, Department of Medicine NYU Grossman School of Medicine New York, NY United States; 3 Department of Rehabilitation Medicine NYU Grossman School of Medicine New York, NY United States; 4 Leon H Charney Division of Cardiology NYU Grossman School of Medicine New York, NY United States; 5 Department of Rehabilitation Medicine, Department of Medicine NYU Grossman School of Medicine New York, NY United States; 6 Division of Cardiology Department of Medicine NYU Long Island School of Medicine Mineola, NY United States; 7 Department of Medicine Division of Cardiology NYU Long Island School of Medicine Mineola, NY United States; 8 Division of Cardiology NYU Langone Hospital Long Island Mineola, NY United States; 9 Section of General Medicine Yale University School of Medicine New Haven, NY United States; 10 Yale University School of Medicine New Haven, CT United States; 11 Cornell Tech and Weill Cornell Medicine New York, NY United States; 12 Department of Medicine University of Massachusetts Medical School UMass Memorial Medical Center Worcester, MA United States; 13 Reynolds Section of Geriatric Medicine University of Oklahoma Health Sciences Center Oklahoma City, OK United States; 14 Department of Population Health NYU Grossman School of Medicine New York, NY United States

**Keywords:** mobile health, cardiac rehabilitation, clinical trial, rehabilitation, cardiology, heart disease, ambulatory care, mHealth, health outcomes, older adults

## Abstract

**Background:**

Participation in ambulatory cardiac rehabilitation remains low, especially among older adults. Although mobile health cardiac rehabilitation (mHealth-CR) provides a novel opportunity to deliver care, age-specific impairments may limit older adults’ uptake, and efficacy data are currently lacking.

**Objective:**

This study aims to describe the design of the rehabilitation using mobile health for older adults with ischemic heart disease in the home setting (RESILIENT) trial.

**Methods:**

RESILIENT is a multicenter randomized clinical trial that is enrolling patients aged ≥65 years with ischemic heart disease in a 3:1 ratio to either an intervention (mHealth-CR) or control (usual care) arm, with a target sample size of 400 participants. mHealth-CR consists of a commercially available mobile health software platform coupled with weekly exercise therapist sessions to review progress and set new activity goals. The primary outcome is a change in functional mobility (6-minute walk distance), which is measured at baseline and 3 months. Secondary outcomes are health status, goal attainment, hospital readmission, and mortality. Among intervention participants, engagement with the mHealth-CR platform will be analyzed to understand the characteristics that determine different patterns of use (eg, persistent high engagement and declining engagement).

**Results:**

As of December 2021, the RESILIENT trial had enrolled 116 participants. Enrollment is projected to continue until October 2023. The trial results are expected to be reported in 2024.

**Conclusions:**

The RESILIENT trial will generate important evidence about the efficacy of mHealth-CR among older adults in multiple domains and characteristics that determine the sustained use of mHealth-CR. These findings will help design future precision medicine approaches to mobile health implementation in older adults. This knowledge is especially important in light of the COVID-19 pandemic that has shifted much of health care to a remote, internet-based setting.

**Trial Registration:**

ClinicalTrials.gov NCT03978130; https://clinicaltrials.gov/ct2/show/NCT03978130

**International Registered Report Identifier (IRRID):**

DERR1-10.2196/32163

## Introduction

Among older adults with ischemic heart disease (IHD), participation in ambulatory cardiac rehabilitation (CR) remains low despite decades of evidence about its benefits. Recent estimates suggest that fewer than two-thirds of eligible patients are referred, and even among those referred, only half attend the first session [1-3]. In addition to barriers faced by the general population (eg, limited facilities, competing time demands, high out-of-pocket costs, and prolonged wait time for space), older adults face additional barriers including lack of transportation, physical limitations, and sensory impairments that make it especially difficult to use existing CR paradigms [4,5]. Therefore, although older adults may have the greatest potential to benefit from CR because of their higher risk of adverse IHD-related sequelae, they are also the least likely to participate [4,6,7].

Mobile health CR (mHealth-CR) for IHD, which involves the delivery of rehabilitation via portable electronic devices, has proliferated rapidly in recent years [5,8,9]. mHealth-CR programs differ but typically include exercise documentation, hemodynamic assessment, video education, and electronic communication with an exercise therapist; these may be standalone components or adjunct to traditional ambulatory CR programs [5,9]. Although mHealth-CR has the potential to increase engagement by reducing participation barriers, it remains largely untested among the older adult population. It is therefore unclear what proportion of older adults with IHD (who may benefit the most) are able to engage with mHealth-CR and whether mHealth-CR leads to better outcomes than usual care in this population. In this paper, we describe the rehabilitation using mobile health (mHealth) for older adults with IHD in the home setting trial, which we designed to address this knowledge gap.

## Methods

### Overview

The rehabilitation using mHealth for older adults with ischemic heart disease in the home setting (RESILIENT) trial (NCT03978130) is recruiting 400 participants with a hospital visit for IHD at 3 academic medical centers: the original 2 sites were New York University (NYU) Langone Health (New York, New York) and Yale New Haven Health (New Haven, Connecticut). The first participant was enrolled on September 1, 2020. A third site, University of Massachusetts (Worcester, Massachusetts), was added in March 2021 to enhance recruitment. For NYU Langone Health, participants are being enrolled at both the NYU Langone Medical Center (New York, New York) and the NYU Langone Hospital–Long Island Hospital (Mineola, New York). NYU Langone Health serves as the coordinating center for both study administration and data management. The primary objective of RESILIENT is to test whether mHealth-CR improves functional capacity, as measured by the 6-minute walk test (6MWT), compared with usual care. We hypothesize that 6MWD (6-minute walk distance) will show significant improvement among participants receiving the study intervention, compared with participants in the usual care arm. RESILIENT was designed using pragmatic trial principles [10], including broad eligibility, the use of existing staff (exercise therapists) to deliver the study intervention, and the inclusion of outcomes (eg, physical function, goal attainment, and quality of life) that have been repeatedly cited as important by older adults [11,12]. There are two study visits: the baseline visit will occur within 4 weeks of hospital discharge and the follow-up visit will occur 3 months after baseline. We chose 3 months for the duration of the study intervention to match the duration of typical CR programs and evaluate whether mHealth-CR promotes early functional recovery.

### Eligibility Criteria

The phenotype of interest for RESILIENT is IHD, which is operationalized as a hospital visit for either acute myocardial infarction (AMI) or coronary revascularization (percutaneous coronary intervention [PCI] or coronary artery bypass graft). We chose the hospital visit as the time of enrollment for two reasons: first, previous research has demonstrated that a serious medical illness or procedure can serve as a *motivational moment* for patients to adopt healthier lifestyles [13,14]; second, deconditioning often accompanies either hospital admission or procedural recovery [15]. As our focus is on understanding mHealth efficacy in older adults, only patients aged ≥65 years are eligible. The exclusion criteria (Textbox 1) were designed to minimize the risk of adverse events (eg, falls with exercise) and ensure that participants can comprehend the study intervention.

**Eligibility criteria for the rehabilitation at home using mobile health in older adults after hospitalization for ischemic heart disease trial.**

**Inclusion criteria**

- Age ≥65 years
- Hospital visit for either acute myocardial infarction or coronary revascularization (percutaneous coronary intervention [PCI] or coronary artery bypass graft)

**Exclusion criteria**

- Nonambulatory or regular use of walker for ambulation
- Moderate or severe cognitive impairment—defined as cognitive impairment that interferes with daily function
- Unable or unwilling to consent
- PCI-related groin hematoma that precludes brisk walking
- Incarcerated
- Unable to use mobile health software in English or Spanish
- Severe osteoarthritis or joint replacement within the last 3 months
- Parkinson disease or other progressive movement disorder
- Projected life expectancy of <3 months
- Clinical judgment concerning other safety or nonadherence issues
- Adverse event during the screening 6-minute walk test (drop in systolic blood pressure ≥15 mm Hg, chest pain, and ventricular arrhythmia)

### Screening and Randomization

Participants are identified through daily electronic health record (EHR) screening of hospital lists with the index condition of AMI, elective PCI, or coronary artery bypass graft. Eligible cases are reviewed by a study physician (JAD, KM, SIC, LK) to ensure they meet the study criteria and are not a false-positive screen (eg, takotsubo cardiomyopathy may be screened by biomarkers, but this does not meet the phenotype of IHD). Potential participants are initially approached while still in hospital whenever possible or, if this is not feasible owing to off-hours procedures, within 48 hours of discharge. The baseline assessment is scheduled within 2 weeks of hospital discharge.

**Figure 1:**
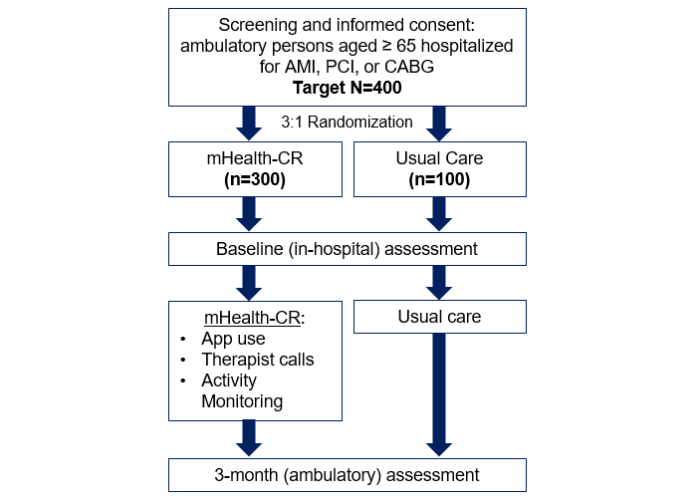
Rehabilitation using mobile health for older adults with ischemic heart disease in the home setting (RESILIENT) study design overview. Participants will be randomized in a 3:1 manner to receive mobile health cardiac rehabilitation (mHealth-CR) versus usual care. A target of 400 participants will be enrolled to retain 320 with evaluable end points (accounting for 20% attrition between baseline and 3 months). AMI: acute myocardial infarction; CABG: coronary artery bypass graft; PCI: percutaneous coronary intervention.

After informed consent and completion of the baseline assessment, including the 6MWT (performed in-person at the study site), enrolled participants are randomized through a function in REDCap (Research Electronic Data Capture; Vanderbilt University) [16] to either the intervention (mHealth-CR) or control (usual care) arm in a 3:1 ratio, using permuted block randomization with variable block sizes of 4 and 8 (Figure 1). The rationale for 3:1 randomization is based on trial efficiency; more participants are enrolled in mHealth-CR than in usual care to have an adequate sample size to understand daily engagement with the intervention. Randomization is stratified by study site to ensure balance across intervention and control groups, given the likely between-hospital population differences. The randomization code was created by the study statistician (SA), and randomization assignments are given by the study coordinator following the 6MWT completion.

### Study Outcomes

#### Primary End Point (Efficacy)

The primary efficacy endpoint is the change in 6MWD, reflective of functional capacity, as measured by the 6MWT. The 6MWT is performed during the baseline visit and at the 3-month follow-up visit by a blinded clinical assessor (exercise therapist or research nurse). At baseline, blinding is maintained by randomization occurring after 6MWT completion in a separate space to ensure that the 6MWT assessor is not present. During follow-up, the randomization group is not disclosed to the assessor by the study staff, and the study documentation with this information is not accessible by the 6MWT assessor. The concept of walking to measure functional capacity was introduced by Balke [17] in 1963, and 6MWD has been used since the 1980s as a robust and reproducible outcome in patients with comorbid illness [18-20]. Among patients with IHD, 6MWD correlates with several clinically meaningful outcomes, including cardiovascular events [21], hospitalization [22], and death [21,23,24]. Changes in 6MWD frequently used by ambulatory CR programs as a measure of effectiveness in patients with IHD [25]. More broadly, changes in 6MWD have been used as an end point in trials focused on a range of cardiovascular conditions, including IHD, and are associated with outcomes including mortality and hospitalization [19,26-28]. The feasibility and safety of measuring 6MWD in patients with IHD, even during inpatient hospitalization, have been demonstrated by previous studies [29,30].

#### Secondary End Points (Efficacy)

There are 5 prespecified secondary efficacy end points.

#### Goal Attainment

*Goal attainment* is defined as whether a person’s individual functional goals are achieved 3 months after hospital discharge and is measured using a 5-point goal attainment scale (GAS). Using the specific, measurable, achievable, realistic, and timely goal framework [11], the GAS describes the person’s expected level of goal achievement over 3 months, ranging from no change (scored as −2) to much-better-than-expected change (scored as +2). Scales are dynamically set according to a person’s needs, whereas the measurement of attainment is standardized. Goals are ascertained by study research coordinators, all of whom obtained structured training by a physician expert (LAJ) in goal attainment. Goal attainment, through goal-setting, is an especially important outcome in older adults who may begin an intervention with a variety of deficits (therefore necessitating individualized therapy toward realistic goals) [31].

#### Participant-Reported Health Status

Participant-reported health status will be measured using the 12-item Short Form Health Survey (general health status) questionnaire [32] and the Seattle Angina Questionnaire 7 (disease-specific health status) [33]. We have chosen these 2 instruments based on extensive validation and convenience of administration (<5 minutes). Changes between the 2 groups will be compared between baseline and 3 months.

#### Changes in Activities of Daily Living

Changes in activities of daily living (ADLs) are defined as any improvement or worsening in basic ADLs (BADLs) or instrumental ADLs (IADLs) over 3 months. BADLs are basic self-care behaviors, including feeding, toileting, bathing, dressing, transferring, and ambulating [34]. IADLs are activities that allow a person to live independently (eg, food preparation, medication management, transportation, shopping, managing finances, using the telephone, and housekeeping) [34].

#### Hospital Readmission

*Hospital readmission* is defined as an unanticipated overnight stay (including observation) in any hospital within 3 months of discharge. As these data are obtainable via the EHR, we also ascertain readmission events at 6 months and 1 year.

#### Death

*Death* is defined as death from any cause within 3 months of enrollment. Similar to readmission, we also ascertain death at 6 months and 1 year through the EHR. For both readmission and mortality, we acknowledge that we may not capture 6 months’ or 1 year’s events that occur outside of the study health systems, although external data linkages are becoming increasingly common.

#### Implementation End Point (Engagement)

We explicitly designed RESILIENT to enable the study of participant engagement with mHealth (an implementation end point) in addition to the efficacy end points. Our main measure of engagement is the weekly percent completion of the mHealth-CR program. Completion of mHealth-CR analyzed at weekly intervals allows us to determine distinct engagement trajectories throughout the 3-month study period. Weekly engagement is measured as the fraction of the following 11 elements completed each week: (1-7) daily entry of exercise data and relative perceived exertion (RPE); (8) completed weekly phone calls with exercise therapists; (9) at least one electronic communication with an exercise therapist; (10) watching an educational video (which varies by week); and (11) at least one home blood pressure (BP) measurement.

### Study Intervention

Study participants randomized to the intervention (mHealth-CR) arm receive three components: (1) mHealth-CR software, (2) communication with an exercise therapist (in-hospital assessment or counseling followed by regular communication postdischarge), and (3) a wearable activity monitoring device. These components are designed to work in concert.

#### mHealth-CR Software

**Figure 2:**
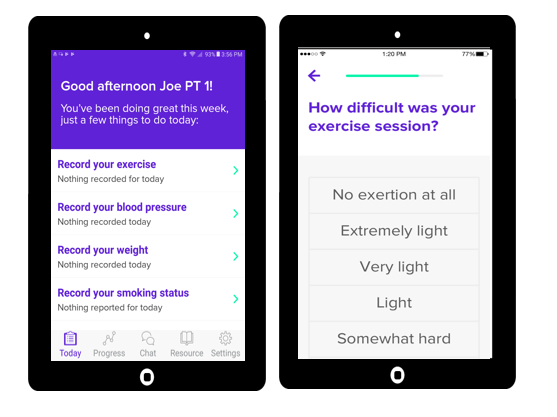
Study intervention. The intervention includes commercially available mobile health cardiac rehabilitation software from Moving Analytics where participants record their physical activity and self-rate difficulty of their exercise session. This is coupled with passive activity monitoring and weekly phone calls with an exercise therapist for a duration of 3 months.

We have partnered with Moving Analytics, which has developed a commercial software platform to deliver mHealth-CR on portable electronic devices (Figure 2). To obviate barriers to portable electronic device ownership, participants will receive a tablet computer (Samsung Galaxy) with mHealth-CR software for the duration of the trial. Devices have cellular capability; therefore, home Wi-Fi access is not required. The software includes four components: (1) participant data entry of exercise duration and RPE; (2) a *chat* function where participants can communicate questions or concerns about symptoms to the exercise therapist (this is checked daily during weekdays); (3) weekly series of educational videos that are focused on secondary cardiovascular event prevention, addressing the following topics—introduction, understanding emotions, exercise guidelines, managing medications, having a heart-healthy diet, stress reduction, and smoking cessation; and (4) physiological measurement with an assessment of BP, heart rate, and physical activity. Participants are instructed by the exercise therapist to use the mHealth-CR app for at least 5 days each week. Training includes education on BP cuff use (cuff placement, seating position, and arm support). The same cuff (Omron HEM-9200T) is used for all the participants. Training also includes how to charge the tablet, how to enter the tablet, how to restart and switch the tablet on or off, how to close all windows, and how to return to the *home* page. All participants are also instructed to log-in to Moving Analytics with the research coordinator present and practice entering activity information. The software is available in English and Spanish. Engagement metrics (daily log-in, activity time on the app, number of exercise sessions logged, duration of exercise, and average weekly minutes of exercise) are captured as a component of the platform and can be analyzed retrospectively.

#### Communication With the Exercise Therapist

The intervention arm participants meet the therapist immediately after the baseline interview. This visit includes education on cardiac risk factor management, assessment of baseline functional status, and an introduction to the mHealth-CR software. A personalized exercise program has been designed and includes alternating aerobic exercise (walking and stair climbing) and low-level isometric resistance training (upper body strength exercises using elastic bands). Participants are recommended to exercise for at least 5 out of 7 days per week, with an ideal goal of 150 minutes per week of moderate-intensity exercise [35]; however, recommendations are tailored based on functional limitations. The therapist identifies potential barriers to this plan and develops mitigation strategies. Given our target population (age ≥65 years), the therapist also assesses home safety during the baseline interview to engage the participant in removing fall hazards at home, and if there is an additional concern, they determine eligibility for a home physiotherapist evaluation after discharge; a similar safety assessment has been successfully implemented in the Strategies To Reduce Injuries and Develop Confidence in Elders study [36]. After this baseline visit, home exercise intensity is rated daily by participants on the mHealth-CR software, using RPE on the Borg Scale (target range 11-14) [37]. The exercise therapist then makes phone contact with the participants in week 1 and weekly (by phone) for the remainder of the study. Exercise recommendations are titrated during calls based on self-reported RPE and a review of activity data. As traditional ambulatory CR is currently the standard of care, participants will also receive a 1-page document containing information about traditional ambulatory CR, including the local facility phone number and recommendations to discuss with their cardiologist. In the interest of a pragmatic trial, referral to traditional ambulatory CR is not mandated for all participants but is left to the discretion of the treating inpatient or outpatient cardiologist (dependent on local practice patterns). Attendance to these programs is captured at 3 months by participant interviews and verified by an EHR review.

The intervention follows the United States’ *Physical Activity Guidelines for Americans, second edition* [38], which has been endorsed by the American Heart Association. The specific guidance is as follows:

- At least 150 minutes of moderate-intensity exercise per week, or 75 minutes of vigorous-intensity aerobic physical activity, or an equivalent combination. Activity should be spread throughout the week (eg, 5 sessions of 30 minutes each).
- If 150 minutes of moderate-intensity activity is reached, participants will be encouraged to increase to 300 minutes of activity.
- Muscle-strengthening activities take place at least 2 days per week. For purposes of the RESILIENT trial, participants are provided with elastic resistance bands and trained on their use at the time of the baseline visit. They are trained to perform upper and lower body exercises using these bands. Each participant is provided with 3 levels of resistance bands to allow for progression and to be able to adjust resistance for the various exercises.
- For those unable to achieve at least 150 minutes of moderate-intensity exercise (eg, owing to functional limitations), lower exercise targets are adapted as endorsed by the guidelines. The principle of move more, sit less will is also recommended.
- Balance training is also incorporated into the treatment plan, as recommended by the guidelines, as they pertain to older adults.

Exercise therapists for the trial have at least a master’s level training in exercise therapy. As clinically trained professionals, they may adapt the intervention based on individual study participants’ physical or sensory limitations or specific rehabilitation needs. This concept is similar to traditional rehabilitation, in which the intervention is individualized.

#### Wearable Activity Monitoring Device

Participants are provided with a Fitbit Inspire or Fitbit Inspire 2 wearable wrist device (Fitbit Inc). This is a commercially available product that measures physical activity based on the number of steps per day. Activity is categorized (based on step count) as sedentary or mildly, moderately, or vigorously active. Heart rate information is also collected by the Inspire 2 model, which was adopted after the first 26 participants. Data are automatically uploaded daily to Moving Analytics and are viewable by both the study participant and the exercise therapist. Weekly phone calls with the exercise therapist include a review of activity data, including the percentage of time spent for each category and total daily step count.

### Usual Care

In accordance with current guidelines [2], participants in both study arms receive information about ambulatory CR at the time of hospital discharge, with the phone number of the program at their respective hospital and guidance to discuss this option with their cardiologist. Therefore, receipt of the mHealth-CR intervention does not preclude participants from attending traditional ambulatory CR. However, in the interest of a pragmatic clinical trial, we do not provide additional incentives (eg, transportation to ambulatory CR) that may further reduce traditional barriers. Similarly, we do not mandate referral to ambulatory CR at the time of hospital discharge because this does not reflect current care patterns at study sites (in current practice, a referral is performed by the outpatient cardiologist). Attendance at traditional CR is captured at the 3-month follow-up visit.

### Study Visits

All participants undergo a baseline visit and a 3-month ambulatory visit, led by a research coordinator who measures the elements listed in Table 1. The baseline visit, which lasts up to 2 hours, occurs either in the hospital or within 2 weeks of hospital discharge. The follow-up visit lasts up to 1 hour. Between visits, all participants undergo telephone assessments of BADLs and IADLs at 1 and 2 months to capture dynamic changes in these measures. Participants in the intervention arm also receive a baseline visit by an exercise therapist (up to 1 hour), regular phone calls (20 minutes), mHealth software, and a Fitbit activity monitoring device. Phone calls follow a structured template to ensure consistency of the study intervention. All study participants receive a US $25 ClinCard payment after completion of the baseline visit and an additional US $90 ClinCard payment after completion of the 3-month follow-up visit.

**Table 1:** Timeline for study participants.

Study arm	Baseline (in hospital)	Home activities	3 months (ambulatory)
Intervention and control arms^a^	In-person assessment Demographics Height, weight, blood pressure 6MWT^b^ Health status (SF-12^c^ and SAQ-7^d^) Activities of daily living Cognition (MOCA^e^) Goal attainment scaling (GAS^f^) Depression (PHQ-9^g^) Frailty elements^h^ Chart abstraction Comorbidities, medications, and laboratory values	Monthly activities of daily living assessment	In-person assessment Weight and blood pressure 6-6MWT Health status (SF-12 and SAQ-7) Activities of daily living Goal attainment scaling (GAS) Depression (PHQ-9) Frailty elements Hospital readmissions Chart abstraction Hospital readmission (verification)^i^ and attendance at traditional cardiac rehabilitation
Intervention arm	Exercise therapist assessment Education on cardiac risk factor management Ascertainment of home environment and mobility barriers Introduction to the mobile health–cardiac rehabilitation software platform Personalized exercise plan	Daily therapist-directed activity (walking and upper extremity resistance training) Daily mHealth data entry Weekly therapist phone call (counseling or activity review) Weekly video education Weekly blood pressure Fitbit activity tracking and review	System Usability Scale

^a^Intervention and control participants will also receive referral to traditional (ambulatory) cardiac rehabilitation at hospital discharge but not mandated or facilitated attendance of first visit. Usual first ambulatory cardiac rehabilitation visit at New York University and Yale takes place within 4 weeks.

^b^6MWT: 6-minute walk test (this will be performed by a blinded research nurse).

^c^SF-12: 12-item Short Form Health Survey.

^d^SAQ-7: Seattle Angina Questionnaire 7.

^e^MOCA: Montreal Cognitive Assessment.

^f^GAS: goal attainment scale.

^g^PHQ-9: Patient Health Questionnaire 9.

^h^On the basis of the 3/5 criteria: unintentional weight loss, weak grip strength (dynamometer), exhaustion, slow gait, and low physical activity.

^i^Hospital readmission will also be ascertained at 6 and 12 months through electronic health record review.

Treatment fidelity is monitored based on principles outlined by the National Institutes of Health’s Behavior Change Consortium (Multimedia Appendix 1) and as described in a review by Borrelli [39]. Specifically, encounters for the first 50 intervention participants are audiotaped and rated by 2 study investigators (JAD and AS) using a structured tool to prevent protocol deviations by exercise therapists. After the first 50 participants, we will review a random sample of 20% of audiotaped encounters. If an individual exercise therapist falls below the a priori performance criterion (ie, a rating below the midpoint of the structured tool) based on an ongoing review of this random sample, the individual remediation will take place through a 1:1 feedback session with an expert in behavioral interventions (AS), who is part of the study team. In addition, a return to 50% monitoring may be warranted. The components evaluated include (1) length of encounter, (2) number of elements covered (eg, review of exercise activity, review of log-in frequency, addressing barriers to activity, and planning exercise for the next week), and (3) nonspecific factors (empathy and communication style) that may influence the success of the intervention.

### Study Management

The Steering Committee consists of the principal investigator (JAD), a biostatistician (SA), and coinvestigator (AS). Moreover, 3 data safety monitoring board (DSMB) members (2 cardiologists and 1 biostatistician) have also been appointed by the National Institute on Aging. The DSMB meets biannually to review recruitment and monitor participant safety.

### Participant Safety

The safety end point includes (1) fall-related injury (operationalized as any fall requiring acute medical care); (2) hospitalization for acute coronary syndrome; and (3) hospitalization for unstable arrhythmia. Separately, among intervention participants, study staff monitor potential exercise-related adverse events on an ongoing basis. Points of contact include weekly phone calls with the exercise therapist and electronic communication via the mHealth app, which is checked daily. All adverse events are reported to the principal investigator and the DSMB. To reduce the likelihood of these events, participants complete the baseline 6MWT before randomization; if any adverse event occurs during the 6MWT (eg, a drop in systolic BP ≥15 mm Hg, chest pain, or ventricular arrhythmia), participants are deemed ineligible for the trial. Other exclusion criteria (severe osteoarthritis, recent joint replacement, and moderate or severe cognitive impairment) are also intended to minimize risk. Previous studies on home-based CR have reported that adverse events are uncommon [40,41].

### Statistical Analysis

#### General

Statistical comparisons will be performed after enrollment of the full study sample, using 2-sided significance tests and 2-sided CIs; no interim comparative analyses are planned. We will begin all analyses with descriptive summary statistics and graphical displays of all variables, with attention to assessing balance in these characteristics by study group assignment and by assessing the distribution of variables relevant to the choice of statistical tests.

#### Primary End Point Analysis

We will assess the difference in 6MWD by calculating difference scores for each participant and comparing the intervention and usual care groups with independent group *t* tests (2-tailed), allowing for unequal variances. We will also regress 3-month 6MWD on a binary indicator of the treatment group, with adjustment for a baseline 6MWD and the stratification factor (enrollment site). Although randomization should obviate the need for additional adjustment, we will explore whether adjustment for participant-level characteristics (eg, demographic factors or referral to ambulatory CR) is necessary, using the change-in-estimate criterion. We realize that engagement with the mHealth-CR program may also affect attendance and engagement with structured ambulatory CR programs. We intend to explore this as a mediator of the effect of the assignment to the intervention arm. We will use structural equation models to estimate the direct and indirect effects of mHealth, where the direct effect is that of mHealth-CR on the 6MWD, and the indirect effect is mediated through the attendance for structured ambulatory CR.

#### Secondary End Point Analysis

Analysis of secondary efficacy end points will proceed in a similar fashion to that of the primary end point.

For *goal attainment,* goals are set with participants at baseline, and *goal attainment* will be assessed at 3 months using a score ranging from −2 to +2 for each participant. To preserve the ordinal nature of the data, we will calculate a median GAS score and then compare the treatment and control groups using a Wilcoxon–Mann–Whitney rank-sum test [42]. We will also compare the percentage of participants in the treatment and control groups who met their expected level of goal attainment (defined as a score of 0, +1, or +2) using a chi-square test.

Health status at 3 months will be assessed using linear regression with adjustment for baseline levels and a binary treatment indicator; if necessary, health status scores will be log-transformed to improve the approximation to normality. ADLs (BADLs and IADLs) will be assessed using longitudinal models for monthly scores, with indicators to incorporate time, a binary indicator of treatment group, and patient-level random effects to accommodate repeated assessments within individuals; if monthly scores are not approximately normally distributed, suitable transformations will be sought. Hospital readmission and death will be evaluated using Kaplan-Meier estimates, tested with log-rank statistics, and investigated using Cox proportional hazard models with adjustment for confounders if necessary. As with the models for the primary end point, in each model described, we will assess the need for adjustment for confounders using the change-in-estimate criterion.

#### Engagement Analysis

Among participants offered mHealth-CR, we will conduct latent class analysis to identify engagement profiles and explore whether these factors indicate membership in a class; these models will use maximum likelihood estimation, implemented with the iterative expectation–maximization algorithm, to identify a latent class solution for the set of indicators. We will evaluate the model fit using the *G^2^* statistic and compare models with the likelihood-difference test for nested models and the Akaike and Bayesian information criteria for nonnested models. Although we have identified four potential classes a priori (sustained engagement, disengagement, re-engagement, and deteriorating engagement), we will use the parametric bootstrap likelihood ratio test to select the optimal number of classes supported by the data in conjunction with the Akaike information criteria and Bayesian information criteria. The output of the model will be a set of *item-response* probabilities giving the likelihood of a particular characteristic within each latent class and a set of posterior predicted probabilities of latent class membership; uncertainty in predicting class membership will be summarized using the odds of correct classification diagnostic tool.

Although the engagement analysis is largely exploratory (given the paucity of data on mHealth-CR engagement), guided by literature related to engagement in other technologies, we will test whether the trajectory classes differ based on the following characteristics: age (≥80 years), sex, race or ethnicity, comorbidity burden (≥2 chronic medical conditions), frailty, social support (based on living alone), and depressive symptoms (based on Patient Health Questionnaire 9).

### Power Considerations

We designed our sample size to detect a clinically meaningful difference between treatment arms in our primary efficacy end point, which is the change in the 6MWD from baseline to 3 months. A recent meta-analysis [25] found an average difference in the 6MWD of approximately 60 meters before and after traditional CR. Perera et al [43] determined that this degree of change in the 6MWD is clinically meaningful based on its relationship to other health status measures. However, Minneboo et al [27] also estimated an improvement in the 6MWD in a control group by approximately 35 m. Therefore, we require adequate power to detect a difference among groups with <25 m in change in the 6MWD (ie, the difference between a 60-m improvement in the intervention arm and a 35-m improvement in the usual care arm); this is the same amount estimated by Gremeaux et al [44] as a minimal clinically important difference in patients with IHD. Assuming a conservative SD estimate of 60 m [25], 320 participants provide approximately 90% power to detect a difference among groups of 25 m, using a 2-sided, 0.05-level test; there is 80% power to detect a difference as small as 22 m. This sample size also provides ≥80% power to detect reasonable effect sizes for the secondary end points. On the basis of a projected attrition rate of 20% dropout in each arm, our target sample size is 400 participants, who will be randomized in a 3:1 allocation. Presuming 20% attrition, this would result in approximately 320 participants with evaluable end points (240 allocated to the intervention arm and 80 allocated to the usual care arm).

### Study Response Owing to the COVID-19 Pandemic

New York was the first epicenter in the United States of the COVID-19 outbreak, with the first case being reported on January 3, 2020. Several weeks later, RESILIENT was closed to new participant enrollment by a university-wide mandate applicable to all clinical trials not related to COVID-19. The NYU Langone Main campus opened to enrollment on June 1, 2020, whereas NYU Langone Hospital—Long Island and Yale New Haven Health opened later (July 27, 2020, and July 20, 2020, respectively). Despite reopening, recruitment remained slow during the remainder of 2020 owing to a combination of slow resumption of normal clinical activities (eg, elective PCI scheduling), lower than expected hospital admissions for AMI, and patient fears of returning to the medical center for study visits.

In response to the pandemic, we made several changes to the original protocol. First, as originally designed, much of the mHealth-CR platform relied on recommendations to walk to achieve physical activity targets. This walking typically occurred outdoors or in large indoor spaces (eg, shopping malls); however, during the pandemic, many participants expressed fear of COVID-19 infection through being in public. Accordingly, with DSMB approval, we provided participants with access to several home exercise videos developed by an exercise therapist affiliated with the study (Dr Patrice Hazan). These videos are assigned by study exercise therapists weekly and include warm-ups, three levels of aerobic workouts (beginner, intermediate, and advanced), and stretch routines. Second, participants are not offered cost transportation reimbursement for the baseline (if occurring within 2 weeks of discharge rather than while in hospital) and 3-month study visits, in addition to the regular reimbursement for participation, to alleviate fears of needing to take public transportation. Finally, the University of Massachusetts was added as a study site to accomplish our recruitment goal.

### Ethics Approval

The study operates on a single Institutional Review Board mechanism (sIRB), and was approved by the NYU School of Medicine Institutional Review Board. The NYU Institutional Review Board study number is 18-02017 for RESILIENT.

## Results

As of December 2021, the RESILIENT trial has enrolled 116 participants. Enrollment is projected to continue until October 2023. The trial results are expected to be reported in 2024.

## Discussion

The RESILIENT study will evaluate whether mHealth-CR improves functional mobility in older adults with IHD and a range of secondary outcomes, including goal attainment, health status, and hospital readmission. To our knowledge, RESILIENT is the largest trial to date for mHealth-CR in an older adult population. We designed this study in light of an aging US population that faces many current impediments to attending traditional ambulatory CR, including transportation barriers and physical impairments, coupled with widespread dissemination of mobile technologies that enable the delivery of CR at home.

Despite the promise of mHealth-CR, definitive evidence of its efficacy among older adults is lacking. Although a recent systematic review reported that mHealth-CR programs led to similar functional mobility and better adherence compared with traditional ambulatory CR programs, most trial participants were <65 years of age [45]. Furthermore, although mobile technology use has increased among the older adult population, it still lags considerably compared with younger patients, and there are many residual barriers to technology adoption, including utility cost (frustration with technology and resistance to change), physical limitations (vision impairment and arthritis), and cognitive challenges (poor memory and impaired reasoning) [46]. Any of these barriers may preclude successful patient engagement with mHealth-CR and limit its effectiveness in older adults. Similar factors may also lead to early termination of mHealth-CR even after successful initial engagement, a phenomenon that has been documented with other mHealth interventions targeted at physical activity [47]. Additional priorities for older patients (eg, clinical visits with multiple specialists) may also compete for patients’ time and limit the number of mHealth-CR sessions completed.

With these barriers in mind, we designed RESILIENT as a hybrid intervention in which the use of the mHealth app is supported by a baseline visit and weekly phone calls by an exercise therapist. These clinical encounters serve to establish rapport between the exercise therapist and study participant, evaluate functional limitations and home safety, and address person-specific barriers to technology use. Although the coupling of human intervention with mHealth technology may limit the scalability of the intervention (as the success of RESILIENT is partially dependent on the proficiency of exercise therapists working at the study sites) and may make it difficult to disentangle the effects of human interaction versus the direct benefit of the mHealth platform, we feel that this tradeoff is necessary to provide the intervention with the best chance of success in the context of the target population. We have built-in auditing of these encounters and direct feedback to ensure the fidelity of the study intervention.

Another decision we made in the design of the RESILIENT trial was to enable both intervention and usual care arm participants to receive traditional ambulatory CR. An alternative strategy, which has been adopted by some trials, would have been a head-to-head study of mHealth-CR versus ambulatory CR. However, we feel that denying older adults access to traditional ambulatory CR in light of limited information about mHealth-CR’s efficacy was not in accordance with the standard of care. In the context of our study, mHealth-CR may therefore serve to reinforce behaviors learned in ambulatory CR among those who attend or as a replacement for those who cannot. Accordingly, we will analyze the heterogeneity of the treatment effect among these 2 subgroups.

We have attempted to minimize bias in the RESILIENT trial through randomized treatment allocation, blinded assessment of the primary end point, and a multicenter design that includes a diverse population. However, we acknowledge that there are several potential sources of residual bias. First, as with any clinical trial, there is selection bias as people who agree to enroll are likely to be more motivated than the general patient population, typically with higher health literacy and a lower burden of chronic illness. Second, there is the potential for transfer bias whereby there may be a differential loss to follow-up in the intervention versus control arms. To minimize this possibility, participants in the control arm receive regular calls from the research coordinator to maintain a connection to the study, and the 3-month visit is scheduled on the same day as a clinical encounter whenever possible to minimize barriers to follow-up.

The generalizability of the RESILIENT trial may be limited by factors including a limited number of study sites, availability of the software only in English or Spanish, and use of a proprietary software from a single company. Furthermore, our intervention pairs the expertise of exercise therapists with an mHealth-CR platform, and positive findings should not be construed as the software platform being effective as a standalone product. We designed the intervention to couple in-person contact with the capabilities of mHealth to guide and reinforce healthy behaviors. In our opinion, this pairing of technology with human interaction—especially in an older adult population that may have limited technological proficiency—provides the best chance of success.

In summary, the RESILIENT trial will generate important evidence about the efficacy of mHealth-CR among older adults in domains including functional mobility, health status, and goal attainment. Moreover, patterns of engagement with mHealth-CR (eg, sustained engagement, declining engagement, and persistent low engagement) will be analyzed to understand the characteristics that predict different trajectories. These findings will help in designing future precision approaches to mHealth implementation and in understanding which patients are likely to engage. This knowledge is especially important in light of the COVID-19 pandemic, which has shifted much of health care to a remote, internet-based setting.

## Appendix

Multimedia Appendix 1 National Institutes of Health reporter data.

## Abbreviations

6MWD: 6-minute walk distance
6MWT: 6-minute walk test
ADL: activity of daily living
AMI: acute myocardial infarction
BADL: basic activity of daily living
BP: blood pressure
CR: cardiac rehabilitation
DSMB: data safety monitoring board
EHR: electronic health record
GAS: goal attainment scale
IADL: instrumental activity of daily living
IHD: ischemic heart disease
mHealth: mobile health
mHealth-CR: mobile health cardiac rehabilitation
NYU: New York University
PCI: percutaneous coronary intervention
REDCap: Research Electronic Data Capture
RESILIENT: rehabilitation using mobile health for older adults with ischemic heart disease in the home setting
RPE: relative perceived exertion
