# Supplementary material for: Rehabilitation Using Mobile Health for Older Adults With Ischemic Heart Disease in the Home Setting (RESILIENT): Protocol for a Randomized Controlled Trial
Source: JMIR Res Protoc. 2022 Mar 3;11(3):e32163. doi: 10.2196/32163 (PMC8931649; doi:10.2196/32163)
Supplement: Multimedia Appendix 1 [file resprot_v11i3e32163_app1.pdf]

Search Results Project Details

Share

Back to Search Results

Description

Details

Sub-Projects

Publications

Patents

Outcomes

Clinical Studies

News and More

History

Similar Projects

Rehabilitation at home using mobile health in older adults after hospitalization for ischemic heart disease (RESILIENT)

Project Number
5R01AG062520-02

Contact PI/Project Leader
DODSON, JOHN A

Awardee Organization
NEW YORK UNIVERSITY SCHOOL OF MEDICINE

Description

Abstract Text

Project Summary Participation in ambulatory cardiac rehabilitation (CR) by patients with ischemic heart disease (IHD) remains low. By recent estimates, fewer than two thirds of eligible patients are referred, and fewer than half of those referred participate. Even among those referred, multiple barriers to participation include limited facilities, competing time demands, high out-of-pocket costs, and prolonged wait time. Barriers to CR are particularly high in older adults (age ≥70), due to factors such as physical impairments or transportation barriers, although these patients may simultaneously have the greatest potential to benefit. Mobile health-enabled CR (mHealth- CR) for IHD – which involves delivery of CR via portab electronic devices – has the potential to increase engagement by reducing participation

Thank you for your feedback!

barriers, but it remains largely untested outside of small studies in relatively healthy young persons. It is therefore unclear what proportion of older adults with IHD and barriers to traditional CR are able to engage with mHealth-CR, and whether mHealth-CR leads to better outcomes than usual care. Therefore, we propose **RESILIENT**: Rehabilitation at home using mobile health in older adults after hospitalization for ischemic heart disease. This is a prospective, multicenter, non-blinded randomized clinical trial (with blinded assessment of primary endpoint) to evaluate engagement and outcomes with mHealth-CR among older adults with IHD, identified at the time of acute myocardial infarction (AMI), percutaneous coronary intervention (PCI), or coronary artery bypass graft (CABG). The trial will be conducted at two academic medical centers: NYU School of Medicine and Yale School of Medicine, which collectively serve a diverse patient population and have a track record of successfully recruiting older adults in clinical research studies. We will randomize 400 older adults with IHD to receive mHealth-CR (n=300) or usual care (n=100) for 3 months. Our intervention combines mHealth-CR software, delivered via a tablet device, with baseline counseling and weekly phone calls by an exercise therapist over 3 months. Intervention and usual care groups will also receive a standard referral to ambulatory CR in accordance with guidelines, as well as dynamic assessment of activities of daily living (ADLs). The primary efficacy endpoint is change in functional capacity, assessed by 6 minute walk distance. Secondary efficacy endpoints are goal attainment, health status, ADLs, hospital readmission, and death. The engagement endpoint is defined by weekly completion of mHealth-CR tasks. We hypothesize that mHealth-CR will improve a range of outcomes, and that distinct patterns of engagement will be discerned. The PI for this project (Dr. **Dodson**) is an Early Stage Investigator with a focus on cardiovascular outcomes research among older adults; additional investigators have a wide range of expertise in geriatrics, biostatistics, behavioral science, cardiac rehabilitation, and computer science. The study results could lead to new sustainable and resource-efficient CR strategies among older adults with IHD, and lay the groundwork for a subsequent large multi-center clinical trial.

Public Health Relevance Statement

Thank you for your feedback!

Project Narrative Traditional cardiac rehabilitation for patients with ischemic heart disease (blocked heart vessels) involves supervised exercise and counseling to improve health. Despite the many known benefits of cardiac rehabilitation, many patients don't use it because of multiple barriers (such as transportation, physical limitations, and cost) which are especially high in older adults (age ≥70). Our study will test whether delivering cardiac rehabilitation through a portable electronic device at home (called "mobile health cardiac rehabilitation") improves function and other outcomes in older adults with ischemic heart disease, and also will evaluate whether these patients continue to use the technology over a period of 3 months.

NIH Spending Category

Aging **Behavioral and Social Science** **Cardiovascular** **Clinical Research**

Clinical Trials and Supportive Activities **Comparative Effectiveness Research**

Health Services **Heart Disease** **Heart Disease - Coronary Heart Disease**

Networking and Information Technology R&D (NITRD) **Physical Activity**

Physical Rehabilitation **Rehabilitation**

Project Terms

Academic Medical Centers **Activities of Daily Living**

Acute myocardial infarction **Aerobic** **Age** **Behavioral Sciences**

Biometry **Blinded** **Cardiac rehabilitation** **Cardiology**

Cardiovascular system **Cessation of life** **Clinical Research**

[illegible]

Read More

Data Disease Documentation Education Elderly Electronic Mail

Electronics Enrollment Ethnic Origin Exercise Face Foundations

General Population Geriatrics Goals Guidelines Health

Health Status Heart Block Home environment Hospitalization

Details

Contact PI/ Project Leader

Name
DODSON, JOHN A

Title
ASSISTANT PROFESSOR

Contact
View Email

Other PIs

Not Applicable

Program Official

Name
ROMASHKAN, SERGEI

Contact
View Email

Organization

Name
NEW YORK UNIVERSITY SCHOOL OF MEDICINE

City
NEW YORK

Country
UNITED STATES (US)

Department Type
INTERNAL MEDICINE/MEDICINE

Organization Type
SCHOOLS OF MEDICINE

State Code
NY

Congressional District
12

Thank you for your feedback!

Other Information

FOA
PA-18-345

Study Section
Behavioral Medicine, Interventions and Outcomes Study Section[BMIO]

Fiscal Year
2020

Award Notice Date
15-May-2020

Administering Institutes or Centers
NATIONAL INSTITUTE ON AGING

CFDA Code
866

DUNS Number
121911077

UEI
M5SZJ6VHUH

Project Start Date
15-July-2019

Project End Date
30-April-2024

Budget Start Date
15-May-2020

Budget End Date
30-April-2021

Project Funding Information for 2020

Total Funding
$660,414

Direct Costs
$474,488

Indirect Costs
$185,926

Year	Funding IC	
2020	NATIONAL INSTITUTE ON AGING	$660,414

NIH Categorical Spending

Click here for more information on NIH Categorical Spending

Funding IC	FY Total Cost by IC	NIH Spending Category

Thank you for your feedback!

NATIONAL INSTITUTE ON AGING	$660,414	Aging; Behavioral and Social Science; Cardiovascular; Clinical Research; [illegible]

Sub Projects

No Sub Projects information available for 5R01AG062520-02

Publications

Export

Journal (Link to PubMed abstract)	Authors	Publ Year		
**Mobile health and cardiac rehabilitation in older adults.**				
Clinical cardiology 2020 Feb; 43 (2) 118-126	Bostrom, John; Sweeney, Greg; Whiteson, Jonathan; Dodson, John A	2020		iCite 2
**Gerotechnology for Older Adults With Cardiovascular Diseases: JACC State-of-the-Art Review.**				
Journal of the American College of Cardiology 2020 12 01; 76 (22) 2650-2670	Krishnaswami, Ashok;	2020		iCite 1

View All

Thank you for your feedback!

Journal (Link to PubMed abstract) Authors Publicati Similar CitedBy iCite R

Patents

No Patents information available for 5R01AG062520-02

Outcomes

The Project Outcomes shown here are displayed verbatim as submitted by the Principal Investigator (PI) for this award. Any opinions, findings, and conclusions or recommendations expressed are those of the PI and do not necessarily reflect the views of the National Institutes of Health. NIH has not endorsed the content below.

No Outcomes available for 5R01AG062520-02

Clinical Studies

No Clinical Studies information available for 5R01AG062520-02

News and More

Thank you for your feedback!

Related News Releases

No news release information available for 5R01AG062520-02

History

No Historical information available for 5R01AG062520-02

Similar Projects

No Similar Projects information available for 5R01AG062520-02

Thank you for your feedback!
